# Effects of maximum and minimum offers on reciprocity and trust perceptions during economic decision-making

**DOI:** 10.3389/fcogn.2025.1576987

**Published:** 2025-11-04

**Authors:** Margaret M. Doheny, Nelson A. Roque, Nichole R. Lighthall

**Affiliations:** ^1^Department of Psychology, University of Central Florida, Orlando, FL, United States; ^2^Department of Human Development and Family Studies, The Pennsylvania State University (PSU), University Park, PA, United States

**Keywords:** reciprocity, decision-making, economic game, trust game, fairness

## Abstract

**Introduction:**

Although it is understood that previous betrayals affect future trust decisions, the degree to which this is true remains unclear in terms of frequency and severity. Additionally, it is currently unknown whether this relationship between the frequency and magnitude of received actions and subsequent trust decisions is mirrored when individuals experience acts of generosity. Prospect theory proposes that losses are weighed more heavily than gains, but the comparison between frequent, minor losses or gains and infrequent major losses or gains has yet to be explored.

**Methods:**

The current study (n = 123) utilizes an adapted version of an economic trust game to examine the effects of minimum and maximum offerings on both reciprocations and perceptions of trust. Participants played the game with two partners: one who offered a maximum or minimum offer (extreme) and one who did not (stable), in either a high or low offer condition where all offers from the stable partner were above or below the median amount, respectively.

**Results:**

The results align with prospect theory in that minimum offers had a greater impact on both behavior and perceptions than equivalent gains (maximum offers).

**Discussion:**

This study highlights complexities between trust, reciprocity, and perceptions of fairness, with implications for understanding social behavior in real-world settings.

## 1 Introduction

Trust is a highly complex component of decision-making that is strongly influenced by personal beliefs, previous experiences, and individual differences. Trust refers to the extent to which a person expects a social partner to aid them in reaching an optimal decision or goal (Simpson, 2007) and the belief in a partner's reliability or benevolence when outcomes are uncertain (Chang et al., 2010). Having to depend on someone else can be precarious, especially if there have been instances of betrayal, or no previous interactions at all. Prior actions of social partners affect reciprocity, which is the “giving of benefits to another in return for benefits received” (Molm et al., 2007), including previous acts of trust. Thus trust and reciprocity are interrelated. Individuals often have expectations of others to behave in accordance with social norms of fairness, wherein “fairness” represents an expectation that exchanges should follow equitable principles (Fehr and Schmidt, 2000). For instance, people typically believe that if they treat others fairly, they will be treated similarly in return. When partners do not behave fairly in return, the perceived probability of risk increases, leading to diminished trust (Haas, 2021). Significant betrayal can lead individuals to generalize these negative feelings, ultimately reducing trust in others, especially those with whom they have no prior relationship (Lee and Selart, 2015). The literature supports the idea that when individuals must make decisions in times of uncertainty, they tend to make fair choices to avoid feeling guilty (Pelligra, 2011). In addition, they may be motivated in their decision-making to evade adverse experiences that could end in loss (Hartley and Phelps, 2012). Despite the wealth of research on trust-related decision-making, it remains unclear if generosity and betrayal have similar effects, in opposite directions, on future trust decisions. Specifically, it is unknown whether maximum values positively impact trust at the same rate as minimum values negatively impact it.

### 1.1 Trust-related decision-making: the role of past experiences

The degree to which an individual is impacted by betrayal is greatly dependent on their history with the social partner (Lee and Selart, 2015), but the effect of betrayal by strangers is less well understood. Research related to betrayal trauma theory has demonstrated that both the greater the dependence on another individual and the amount of trust invested in them strongly predicts the degree to which one is negatively impacted by betrayal (Martin et al., 2013). When deciding whether to trust a stranger, there is no record of previous actions with which to assess their trustworthiness (Lee and Selart, 2015). Decisions to trust individuals where we have no track record with become highly dependent on individual differences, baseline levels of trust, and generalizations from previous experiences (FeldmanHall et al., 2018). Individuals with emotional irregularities tend to have lower baselines of trust and experience more prominent negative emotions when betrayed by a stranger (Lee and Selart, 2015). The current study involves gameplay with a stranger; therefore, the only indicator of trustworthiness is the behavior of the partner that is experienced during the game.

### 1.2 Extreme behaviors and valence of outcomes

Research on the consistency of behavior and its subsequent consequences is rather scattered. In everyday encounters, we tend to value those who consistently fulfill trust, but, in reality, behavior is often varied and erratic (Mischel and Peake, 1982). Individuals exhibit more resistance to trust in unpredictable situations or when actions defy expectations (Abbott et al., 1984). Stress resulting from uncertainty is prevalent in social interactions (Starcke and Brand, 2016), which leads to the notion that consistency is preferred. Research demonstrates that the subjective impact of betrayal can exceed financial loss (Bohnet and Zeckhauser, 2004). Instances of betrayal can often cause great shock and ultimately create skepticism of the betrayer (Rachman, 2010). The outcomes of betrayal depend on the severity of the situation and can include damaged or broken trust, grief, rumination, loss of self-esteem, and frustration (Rachman, 2010). Feelings of betrayal can even cause psychological trauma that result in fight-or-flight behaviors toward the betrayer (Freyd, 1999). Other betrayal outcomes can include revoking trust from uninvolved individuals (Lee and Selart, 2015). This generalization of betrayal is thought to reflect a protective strategy to avoid additional harm, and is sometimes permanent (Rachman, 2010). In economic trust games, betrayal can be observed from failure to reciprocate a partner's investment or other loss outcomes that can be attributed to the partners' intentions (e.g., failing to send endowed investment money to a partner who has consistently reciprocated in the past; Lee and Selart, 2015). The impact of betrayal also varies based on the expected value of the outcome (Joskowicz-Jabloner and Leiser, 2013). In other words, if someone has high hopes of a certain outcome and it does not occur, the feelings will be more negatively impactful than if the individual had low expectations. Betrayal occurring prior to the trust game can also cause substantial shifts in behavior that are driven by spite or self-preservation (Martin et al., 2013). Despite this wealth of research, it is unclear if extreme betrayals have a different impact on the likelihood of reciprocation compared to more frequent, lower-level betrayals that result in equivalent losses over time.

There has been much research demonstrating that zero is a special value in relation to behavior commonly known as the “zero effect” (Zhang and Slovic, 2019). However, recent research on the zero effect focuses on consumer behavior, specifically in terms of how offerings of free products affect decisions (Murata et al., 2017). Findings suggest that the outcome of the zero effect is dependent on both context and other values acting as reference points (Palmeira, 2011). Therefore, recent studies investigate the value of zero as a positive option, as opposed to a negative option. This has been demonstrated by Zhang and Slovic, not in an economic context, but in terms of life-saving decisions (zero as an outcome where no one dies). The findings of their study suggest that people tend to give less value to other small nonzero values (values other than absolute zero) when compared to the value of zero (Zhang and Slovic, 2019). This has yet to be investigated in the context of economic decision-making. Specifically, it must be further investigated whether (1) zero dollar values impact trust-related decision-making when all other values are small, and (2) this effect is replicated with a maximum value, with other favorable values as reference points. The current study seeks to examine how maximum and minimum offers affect decisions to reciprocate in a trust game, when all other offerings are similar in value. This is yet to be explored and can provide great insight into how individuals make trust decisions and what types of actions significantly impact social relationships.

### 1.3 Measuring decisions to reciprocate

Factors that influence trust decisions are often examined using economic paradigms such as the trust game (Berg et al., 1995) in which participants make choices based on principles of self-interest and monetary gain (Fehr and Schmidt, 2000). While the original task has been referred to as a “trust game,” it is more accurately a measure of reciprocity, as the central decision concerns whether to return resources previously entrusted by another player, rather than initiating trust. In economic tasks, the definition of trust is often redefined to categorize the probability of reciprocation from the specific social partner (Chang et al., 2010). Within this framework, reciprocity is shaped by perceptions of the partner's trustworthiness and expectations of fairness, which guide whether individuals respond cooperatively or protect their own outcomes (Chang et al., 2010; Johnson and Mislin, 2010). Individual differences, such as lower baselines of trust due to trauma or previous betrayals, motivation to attain a goal, social influences of fairness, and personality traits, can affect one's strategies in economic trust games (Fehr and Schmidt, 2000; Joskowicz-Jabloner and Leiser, 2013; Grecucci et al., 2013). Importantly, rates and levels of reciprocity from one's partner are among the strongest predictors of decision-making in this paradigm, with unfair behavior often eliciting lower reciprocation in return (Chang et al., 2010) The current study adopts Berg et al. (1995) task, maintaining the established “trust game” terminology, while clarifying that our focus is on operationalizing reciprocity behavior, alongside measured perceptions of trustworthiness and fairness expectations.

### 1.4 Prospect theory

A prominent theory in the arena of trust games is prospect theory, which supports the idea that individuals are more focused on loss aversion, and that losses are more impactful than gains (Kahneman and Tversky, 1977). Although a valid application to trust-related decision-making, prospect theory requires further exploration in this context (Barberis, 2012). Recent work utilizing prospect theory as a foundation has shifted from economical models to direct applications to real-world human decision-making scenarios. For example, Gao et al. (2021) tested it on decisions made when commuting, with time saved or lost as the currency, and found that behaviors differ greatly compared to what was found in previous economical tasks. Another recent work used prospect theory as a theoretical foundation for a new model to evaluate the risk of cognitive bias specifically in the domain of renewable energy (Ilbahar et al., 2022). Additionally, many researchers have focused on creating mathematical models to further expand on the basic components of prospect theory to incorporate multi-level decision-making (Leoneti and Gomes, 2021; Chai et al., 2023; Tan et al., 2023; Zhang et al., 2022). Despite these advancements in prospect theory, researchers have called for work that thoroughly evaluates its basic components, as there have been many conflicting findings in terms of evaluations of net vs. gross gains or losses (Harrison and Swarthout, 2023). This still remains unclear; there is no work to date examining differences in decision-making behavior when gains or losses are minor yet consistent, compared to those that are major yet infrequent. By directly looking at how individuals respond to a few large gains or losses vs. consistent but smaller gains or losses, we can get new insights into the foundations of prospect theory.

### 1.5 The present research

In social decision-making with strangers, it is unclear which behaviors play the strongest role in the establishment and maintenance of trust. With this, we are interested in several research questions. First, do individuals favor consistent positive actions, or less consistent, but still favorable actions with rare, grand acts of generosity when these conditions yield the same financial outcome in the long run? Second, do the effects of consistency and outcome magnitude depend on whether the social interaction is considered fair. We will compare the effects of social partners with consistent yet suboptimal behaviors to those who offer more extreme outcomes, representing great generosity or betrayal, when these conditions yield the same financial outcome in the long run.

The current study utilizes the aforementioned literature to create a novel study design to evaluate our research questions. We aim to assess how varying degrees of betrayal impact decisions to reciprocate and perceptions of partner trustworthiness. We created an adaptation of the original trust game paradigm developed by Berg et al. (1995) with a few minor changes in the form of between-subjects conditions and role reversals to examine our central research questions. In the original trust game, participants are typically assigned to the “investor” role, but we assigned all participants to play as the “trustee.” Investors submit a dollar amount, while trustees decide whether to reciprocate or not based on that endowment. In the current study, we are interested in how the dollar amount affects trust decisions that can occur when the participant is the trustee. Additionally, contrary to a typical study including the trust game, we implemented a between-subjects factor of high and low offers and a within-subjects factor of consistent vs. extreme offer conditions. With our design, we seek to juxtapose consistently behaving social partners with partners who are more extreme in their actions. Through this, in the high offer condition, we can examine whether individuals value consistency or “redeeming” actions marked by a few extremely generous offers. In a condition where all offers are subjectively low, we can examine the effect of extreme losses paired with relatively fair offers, vs. consistent, yet unfair behavior.

The present study tested several hypotheses in the areas of reciprocity across all trials and the comparison of trustworthiness ratings to reciprocity.

- **Hypothesis 1a:** Reciprocity across all trials will be less likely in the low condition than the high condition; we expect a significant main effect of condition (high/low) on reciprocity.
- **Hypothesis 1b**: The differences in reciprocation between stable and extreme partners will be greater in the low offer condition compared to the high offer condition. Therefore, we expect a significant interaction between high/low offer condition and extreme/stable partners.
- **Hypothesis 2a:** The likelihood of a reciprocation on the last trial will be greater in the high condition compared to the low.
- **Hypothesis 2b:** Reciprocation on the last trial will be more likely in the stable condition than the extreme condition. We expect to observe an ordinal effect of most likely to reciprocate to least likely to reciprocate as (1) extreme-high, (2) stable-high, (3) stable-low, and (4) extreme-low.
- **Hypothesis 3a:** Post-task perceptions of trustworthiness will mirror the results expected for reciprocation under Hypothesis 1. Specifically, we expect that higher trustworthiness ratings will be associated with more reciprocations across the game, and lower trustworthiness ratings will be associated with fewer reciprocations.
- **Hypothesis 3b:**We expect a significant interaction between high/low offer conditions and extreme/stable partners for trustworthiness ratings.

## 2 Methods

### 2.1 Participants

We based our sample size on Zhang and Slovic's (2019) work on the zero effect, where there were approximately 50 participants in each between-subjects condition. Participants (*n* = 200) were recruited via the online data collection platform Prolific. After reading an explanation of the research, prospective participants could decide whether they were willing to participate. Participants recruitment was limited to those from the United States. Participants had to read and accept the consent form and state that they were 18 years old to proceed to the demographics form and task. Participants received a base pay of $6 and were told that they could receive an additional bonus up to $4 based on a percentage of their earnings across all trials. In reality, all participants were given the maximum bonus, and therefore compensated $10, standard for a 30-min experiment. This compensation cover story, wherein participants are told the bonus amount will be a percentage of earnings across all trials, yet all participants are compensated the same amount regardless of performance, is common with multi-round trust games (Bose and Camerer, 2021). At any time, the participant could close the browser window to end the survey without penalty. As an online study, the trust game paradigm was created in JSPsych and embedded into a Qualtrics survey containing the consent form, the task, follow-up surveys, and a debriefing statement. After excluding participants who either did not complete the entire study or attempted multiple submissions, a total of 123 participants were included in the final analysis. Participants' ages were in the range 19–74 years (*M* = 40.11 *SD* = 13.41). See Table 1 for full participant characteristic information. As noted in Section 3, “Results,” the present study observed interactions of within- and between-subject factors with small-to-moderate effect sizes. *Post hoc* sensitivity analyses indicated that, given the study's final sample size (*n* = 123), the experiment had >80% power to detect these interactions.

**Table 1:** Participant characteristics.

**Characteristic**	**High condition (*****n =*** **58)**		**Low condition (*****n =*** **65)**		**Total (*****n** =* **123)**	
	***n***	**%**	***n***	**%**	***n***	**%**
**Sex at birth**						
Male	31	53.4	34	52.3	65	52.8
Female	27	46.6	31	47.4	58	47.1
**Race**						
Native American/Alaskan	1	1.7	1	1.5	2	1.6
Asian	0	0.0	3	4.6	3	2.4
Black/African American	8	13.8	7	10.8	15	12.2
White	45	77.6	49	75.4	94	76.4
More than one race	4	6.9	5	7.7	9	7.3
**Ethnicity**						
Hispanic/Latino	8	13.8	8	12.3	16	13.0
Not Hispanic or Latino	50	86.2	57	87.7	107	86.9

### 2.2 Trust game

**Figure 1:**
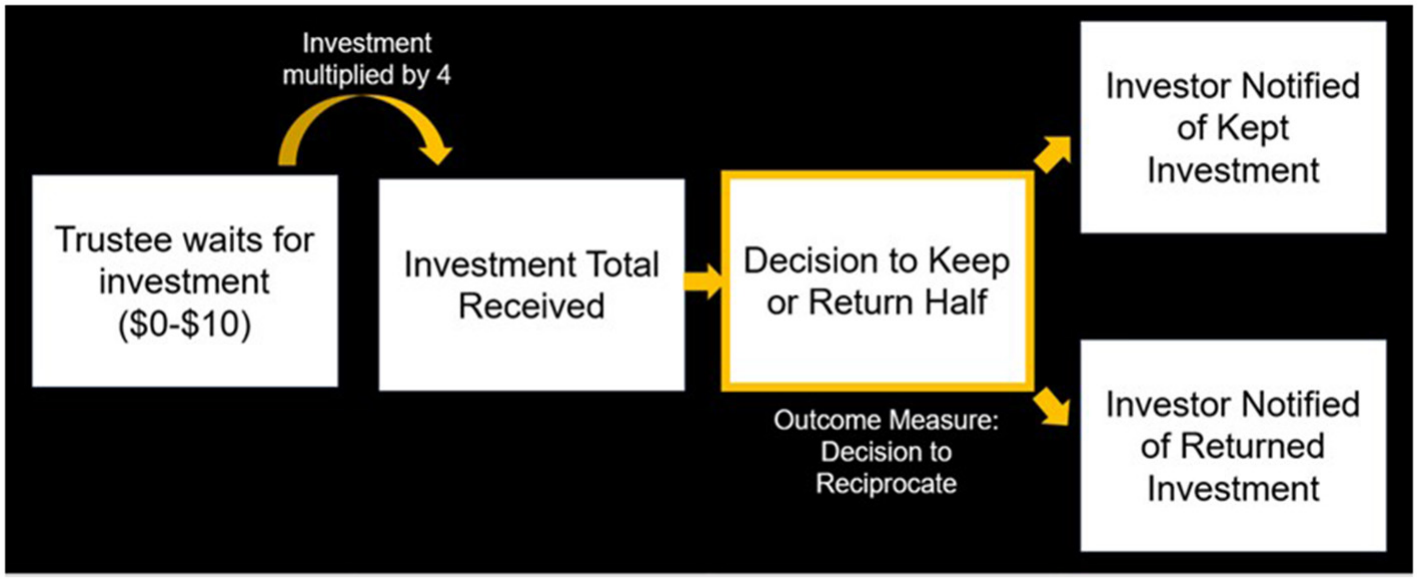
Depiction of one round in the trust game.

Participants completed a multi-round “trust game” experiment adapted from Berg et al. (1995). In the game, there are two roles: investor and trustee. In each round, the investor chose a dollar amount to send to the trustee, which was then multiplied by four. The trustee must decide whether to keep the full quadrupled amount or return half back to the investor (i.e., reciprocate). In the current study's version of the trust game, participants engaged in economic interactions with two different partners. Each partner was actually a computer program, although the participants were led to believe that they were playing with another Prolific user in real time. We added faux loading screens at the beginning of each new partner to create the notion that Prolific was searching for another user. Additionally, we implemented randomized durations for when the partner was “choosing their offer amount.” Participants were notified that their partner can invest any amount from $0 to $10, and that investment would be multiplied by four before being given to them. To observe ecologically valid choices, participants were told that at the end of the experiment, one trial would be selected at random, and they would be paid a bonus amount of a percentage of their earnings from that specific trial (see Figure 1).

We included avatars in our study for the purpose of encountered partner identification in follow-up measures of memory and perceived trustworthiness. Avatars were neutrally-rated, non-social images from the International Affective Picture System by Lang et al. (2008) (see Supplementary material for avatar images). Participants were told that all investors chose an avatar to represent themselves, while trustees remained anonymous. Avatar images were randomized across all conditions for each participant. Participants were randomly assigned to either the “high” or “low” game condition at the beginning of the experiment. Participants had 30 interactions (trials) with each partner. All offers from investors in the “high” condition were equal to, or exceeded, $5. All offers from investors in the “low” condition were equal to, or below, $5. These conditions represented partners whose behavior was relatively more or less trustworthy, respectively.

Participants played with two different partners within their assigned high/low group condition (see Table 2). The order in which participants encountered partners within-subjects was counterbalanced. In the high condition, the extreme-high partner offered at or just above a fair split in 90% of trials (*M* = $7.02; SD = 1.43; range = $5.00–$10.00), with significantly generous offers of the maximum amount of $10 in 10% of trials (pseudo-randomly assigned). The alternate stable-high partner gave offers slightly higher than the modal offer in the extreme-high condition on every trial (*M* = $7.04; *SD* = 1.14; range= $5.00–$9.00), with no extreme offers and the same overall expected value across trials as partners in the extreme-high condition. In the low condition, the extreme-low partner invested at or just below a fair split in 90% of trials (*M* = $2.99; *SD* = 1.29; range = $0.00–$5.00), with significantly unfair offers of $0 in 10% of trials (pseudo-randomly assigned). The alternate stable-low partner gave offers slightly lower than the modal offer in the extreme-low condition on every trial (*M* = $3.00; *SD* = 1.17; range = $1.00–$5.00), with no extreme-low offers and the same overall expected value across trials as partners in the extreme-low condition. With nearly equivalent means, the only component that differed between stable and extreme partners was the range; only extreme partners offered the minimum (low condition) or maximum (high condition). To further support this, *t*-tests demonstrated that there were no significant differences between stable and extreme partner offers in both the high condition (*t* = 0.195, *p* = 0.847), and low condition (*t* = −0.684, *p* = 0.500). Thus, our analysis directly isolated the effects of maximum and minimum offers on participant behavior.

**Table 2:** Between-subject and within-subject conditions and descriptions of roles.

**Partner name**	**Description**
Stable-low	Offers consistently less than a fair split with ***no extreme-low offers*** of $0 (minimum).
Extreme-low	Offers a less than fair split but slightly more than the modal offer in the stable-low condition, with three ***instances of extreme-low offers*** ($0). Overall expected value of offers equal to stable-low condition.
Stable-high	Offers consistently more than a fair split with ***no extreme-high offers*** of $10 (maximum).
Extreme-high	Offers more than a fair split but slightly less than the modal offer in the stable-high condition, with three ***instances of extreme-high offers*** ($10). Overall expected value of offers equal to stable-high condition.

We ensured that the minimum and maximum offers, although randomized through programming, did not tend to occur more or less in specific blocks, which could potentially skew the results. We calculated the number of times extreme offers were given within the first block of both conditions to examine this effect. We conducted independent samples *t*-tests to examine the occurrences of extreme offers for each extreme partner in both high and low conditions for each block. We found no significant differences in the frequency of extreme offers between blocks in either the high or low conditions, confirming that these values were not skewed to occur more or less in different points of the game. Finally, in addressing potential differences in behavior within the first block, we can attribute that to chance based on little to no previous interactions. Participants were actively learning their partner's behavior in the first block and could have been experimenting to see how their partner responded. Partner order was balanced, so order effects are not an issue.

### 2.3 Survey measures

After the task, participants completed a series of personality surveys and follow-up questions. The surveys were implemented to allow for time to pass between the task and the related follow-up questions. In the follow-up, participants were tested on their memory of their partners. They rated their memorability on a 5-point Likert scale (1 = *I definitely did not play with this partner*; 5 = *I definitely did play with this partner*). There were four memory trials in total. Two trials displayed avatars that were encountered, and two were new avatar images (foils). Next, participants rated the subjective trustworthiness of both encountered and foil partners on a scale of 1–100, with a rating of 100 to indicate maximum trustworthiness. Participants had the option of choosing “not applicable” if they did not encounter that specific partner. Finally, all possible combinations of partners were presented in choice trials, wherein participants chose which of the previously encountered partners they would rather play with again if given the chance. Again, there was an option to select “I did not play with at least one of these partners.” These questions were designed to assess the effects of the experimental conditions on memory and preference. Following this, participants were debriefed about how they were not interacting with another human but rather with a computer, and that all participants would be receiving the same maximum bonus.

To determine the validity of our trustworthiness rating measure, we only included participants who gave trustworthiness ratings to both partners they actually played with (*n* = 78). In some cases, participants mistakenly rated the two foils in this section of the series or neglected to rate the two partners in which they encountered. To further confirm the validity, we calculated corrected recognition scores, which measures correct partner identification while accounting for bias to falsely identify foils in the memory assessment (see below). As previously stated, participants had to identify whether they remembered playing with both partners in which they encountered, as well as the two foils on a 5-point Likert scale (1, *I definitely did not play with this partner*; 5, *I definitely played with this partner*). We then calculated a “memory score” for stable and extreme partners, in high and low conditions. We did this by subtracting the average rating of both foils from the rating for each encountered partner. With this, the highest possible score was 4, given that they selected that they definitely played with the encountered partner (5) and that they definitely did not play with both foils (1). The average scores for each encountered partner were 3.49 for stable partners and 3.50 for extreme partners. We then were able to confirm the validity of the trustworthiness ratings due to acceptably good recall for encountered partners. We acknowledge that we had to exclude a large number of participants from the follow-up analyses to maintain the validity of the trustworthiness ratings. Although trustworthiness ratings could have occurred immediately after the conclusion of the 30 trials, we purposely placed our ratings post-survey to examine whether either partner had left a lasting impression on the participant. Therefore, although we were left with a smaller sample size, we believe that the trustworthiness ratings assessed are high in validity.


$$\begin{array}{l} \left(\left(Foil\;1\;Rating\right)\;+\;\left(Foil\;2\;Rating\right)\right)/2\;=\;Foil\;Average.\\ \left(Encountered\;Partner\;1\;Rating\right)\;-\;\left(Foil\;Average\right)\;=\\ Memory\;Score\;Partner\;1.\\ \left(Encountered\;Partner\;2\;Rating\right)\;-\;\left(Foil\;Average\right)\;=\\ Memory\;Score\;Partner\;2. \end{array}$$

### 2.4 Qtest 2.1

The method for executing data analysis for Hypothesis 2 was Qtest 2.1, developed by Regenwetter et al. (2014). Qtest 2.1 is an open-source public domain software written in MATLAB that allows for testing of precise, order-constrained hypotheses with incorporation of heterogeneity. Within Qtest 2.1, Bayesian methods were used to test order-constrained inferences with variable binary data. Hypotheses in Qtest 2.1 must be entered based on likelihood from 0 to 1, starting with the least likely variable being greater than or equal to 0. Since variables must be entered with the option to be equal to another variable in terms of likelihood, we are able to examine exactly how conditions relate to one another in terms of reciprocation. Several models were assessed in Qtest 2.1 to evaluate which was the best fit for the data in terms of theory testing. In Qtest 2.1, we examined the choice to reciprocate on the last trial of each partner. This outcome variable was assessed by high/low and consistent/inconsistent condition. For the outcome variable of reciprocation, we evaluated two hypothetical models and one mixture model, with results reflecting order-constrained Bayesian inferences. Model comparison was conducted using Bayes factors and deviance information criterion (DIC), with posterior predictive checks performed to assess model adequacy (Regenwetter, 2020). Bayes factor tests were run with a Gibbs sample size of 100,000,000 and Bayesian *p* and DIC were run with a Gibbs sample size of 100,000.

The model implemented in Qtest 2.1 was to measure the returns in the final trial in each block, accounting for between-subjects and within-subjects. Hypotheses 2a and 2b were directly tested in Model 2a. Again, we predicted that the low condition would have fewer reciprocities on the last trial than the high condition, and that stable partners were more likely to reciprocate on the last trial. Additionally, we ran the inverse or alternative (Model 2b), and the mixture or null (Model 2c) to confirm our hypothesis and determine the best-fitting model for the data. Below are the order-constrained hypotheses implemented in Qtest 2.1, displaying the conditions in order from least likely to reciprocate (0) to most likely to reciprocate (1).

**Key:** Extreme-high (EH), Stable-high (SH), Extreme-low (EL), Stable-low (SL).

**Model 2a:** 0 ≤ EL ≤ SL ≤ EH ≤ SH ≤ 1; 0 ≤ SL – EL ≤ SH – EH; SH – EH < 1; SL – EL > 0.

**Model 2b:** 0 ≤ SH ≤ EH ≤ SL ≤ EL ≤ 1; 0 ≤ EH – SH ≤ EL – SL; EL – SL < 1; EH – SH > 0 (inverse).

**Model 2c:** Mixture of Hypotheses 2a and 2b (null).

## 3 Results

### 3.1 Model 1: examining reciprocation across all trials and conditions

**Figure 2:**
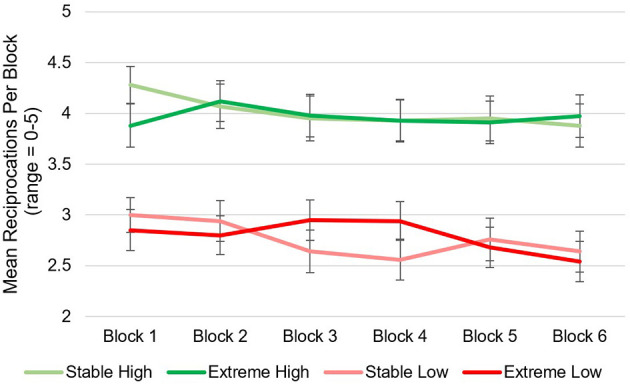
Means and standard errors of the mean of high/low condition, stable/extreme partner, and block (six blocks of five trials) on reciprocation. Means indicate number of reciprocations out of five trials, with minimum 0 (indicating no reciprocations) and maximum 5 (indicating reciprocating on each trial in block).

For Model 1, we hypothesized that reciprocity across all trials would be less likely in the low condition than the high condition and that reciprocity across all trials would be more varied between partners in the low condition compared to the high condition. We calculated the proportion of reciprocation for each participant for each block of five trials. Block-wise decision analysis is commonly used for multi-round trust games (Chang et al., 2010; Li et al., 2017). We then conducted a repeated measures ANOVA to evaluate between- and within-subjects effects on the rate of reciprocity for each of six blocks and examined estimated marginal means. From Figure 2 we can see that the high conditions had more reciprocations on the last trial than the low. Additionally, there appears to be greater variance between partners in the low condition compared to the high condition.

In the repeated measures ANOVA containing a within-subjects effects of extreme/stable conditions and blocks of five trials and a between-subjects effect of high and low condition, there were several significant findings. First, we observe a significant difference in low and high conditions, with a large effect [*F*_(1, 124)_ = 25.78, *p* < 0.001, $\eta_{p}^{2}$ = 0.17], as expected, in that the partners in the high condition received more reciprocations. Additionally, the within-subjects effect of block was significant with a moderate, linear effect [*F*_(1, 124)_ = 12.12, *p* < 0.001, $\eta_{p}^{2}$ = 0.08], depicting changes in reciprocation behavior throughout the trials. Specifically, we see that throughout the course of the game, reciprocations tended to decrease across trial blocks. We additionally observed a significant, quadratic interaction between block and extreme/stable conditions, with a moderate effect [*F*_(1, 124)_ = 7.44, *p* = *0.0*07, $\eta_{p}^{2}$ = 0.06]. Separate *post hoc* tests for block effects in the stable and extreme conditions yielded significant block effects in the stable partner condition only. A small quadratic block effect [*F*_(1, 121)_ = 5.11, *p* = 0.03, $\eta_{p}^{2}$ = 0.04] and moderate linear block effect [*F*_(1, 121)_ = 12.21, *p* < 0.001, $\eta_{p}^{2}$ = 0.09] were observed in this condition. In the extreme partner condition, there were no significant block effects observed (all *p*-values > 0.116). These *post hoc* tests reveal that the stable partner condition was driving the interaction of stable/extreme condition and block. Critically, results for reciprocity by block were not confounded by ceiling or floor effects, as 95% confidence intervals for mean reciprocity by block within individual extreme/stable and high/low conditions did not overlap with the minimum (0) or maximum (5) number of reciprocations per block (see Supplementary Table 1).

### 3.2 Model 2: examining likelihood of reciprocation on last trial by condition

For the second model, we again had two hypotheses. Hypothesis 2a predicted that there would be less reciprocations during the last trial in the low condition than the high condition. Hypothesis 2b postulated that stable partners would receive more reciprocations in the last trial than extreme partners. Both hypotheses were tested in Model 2a, then compared to the inverse (Model 2b) and the null (Model 2c) to determine the best fit. Model 2a was ultimately the best-fitting model due to it having the highest Bayes factor, lowest DIC, and a Bayesian *p*-value close to 0.5 (See Table 3 for Model 2 results). These results support Hypothesis 2a in that the high condition would receive more reciprocations than the low condition. Although this model was the best fit compared to the inverse and null, we received partial support for Hypothesis 2b. In the low condition, the stable partners did receive more reciprocations than extreme partners, but this did not occur in the high condition. The extreme-high partner received slightly more reciprocations than the stable-high partner. However, the difference in the proportion of reciprocations between extreme-high and stable-high conditions was only 1.66%. This lack of difference supports the notion that maximum ($10) amounts do not create a major impact on reciprocation when all offers are above the median amount or “fair.” Finally, as predicted, the stable-low partner received more reciprocations than the extreme-low partner, demonstrating that the minimum ($0) offers have a greater impact on reciprocation than maximum offers. To provide an alternative test for any response bias starting from the beginning, we conducted chi-squared tests on the decision to reciprocate on the first trial. We found no significant association between condition and decision to reciprocate, in both the high condition [$\chi_{\left(1,116\right)}^{2}$ = 2.94, *p* = 0.09] and the low condition [$\chi_{\left(1,130\right)}^{2}$ = 0.62, *p* = 0.43], demonstrating that no prominent response bias was set from the beginning of the trial. We completed this to ensure that reciprocation on the last trial was a valid measure of learned behavior across the entire game.

**Table 3:** Model 2 fit statistics exhibit Model 2a to be the best-fitting model.

**Model**	**Bayes factor**	**DIC**	**Bayesian *p***
**Model 2a**	**6.4330**	**6.0106**	**0.5466**
Model 2b	2.0662	21.9005	0.0008
Model 2c	1.8617	7.3793	0.4354

Results depict little to no difference in reciprocation in the last trial for both high conditions. Great variance is observed between high and low conditions in last choice to reciprocate. Ultimately, extreme-low had the least amount of reciprocation, indicating that minimum offers had a large impact, while maximum offers did not.

Bold text indicates best-fitting model.

### 3.3 Model 3: examining partner trustworthiness ratings across conditions

**Figure 3:**
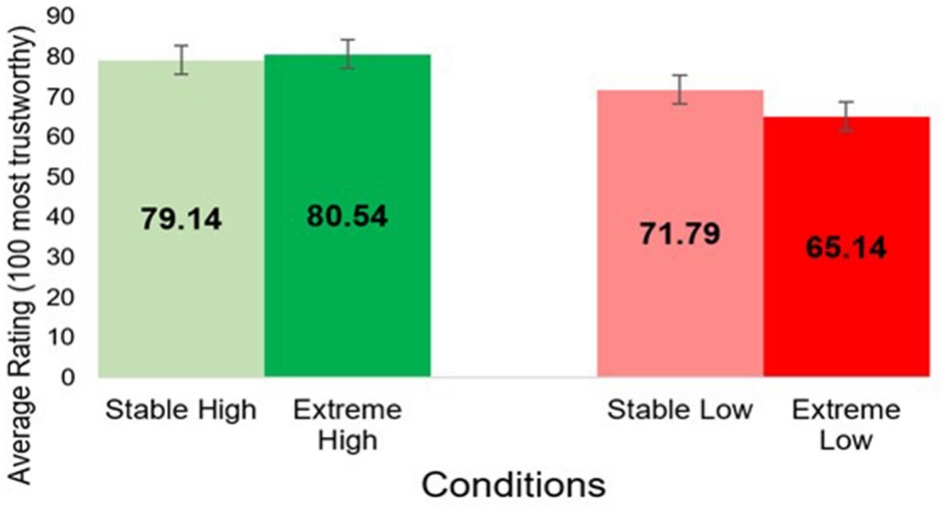
Estimated marginal means for post-experiment trust ratings for encountered partners. Results indicated greater perception of trust for partners in the high condition than low, with extreme offers leading to reduced ratings of trust in the low condition only.

For Hypothesis 3a, we predicted that perceived trustworthiness ratings would mirror the expected results from Hypothesis 1a for reciprocations. We additionally predicted a significant interaction between high/low offer condition and extreme/stable partners with trustworthiness ratings for Hypothesis 3b. We conducted a repeated measures ANOVA to look at within-subject effects of stable/extreme conditions, controlling for high/low conditions with the outcome measure of trustworthiness ratings. When examining trustworthiness ratings, the results indicate significance on the between-subjects effect of low/high condition to a moderate degree [*F*_(1, 76)_ = 6.69, *p* = 0.01, $\eta_{p}^{2}$ = 0.081], demonstrating that trust ratings were lower for partners in the low condition compared to the high condition, mirroring results from Hypothesis 1a. Finally, we observed significance with a moderate effect in the interaction of extreme/stable and high/low conditions [*F*_(1, 76)_ = 6.74, *p* = 0.01, $\eta_{p}^{2}$ = 0.08], mirroring results from Hypothesis 1b. These results show that trustworthiness ratings were significantly different in both high and low conditions and between stable and extreme partners. More specifically, we see higher trustworthiness ratings in the extreme partner in the high condition, but by a negligible amount (1.4). In the low condition, trust ratings were significantly higher for the stable partner. We examined estimated marginal means to visualize average trust ratings for each condition (see Figure 3). It is important to note that these results directly mirror that of Hypotheses 2a and 2b, indicating that perception of partner trustworthiness was reflected in the last choice to reciprocate in the game.

## 4 Discussion

The purpose of this study was to investigate how maximum and minimum offers affect reciprocation behavior in an economic trust game when all other offers are values similar in number but not at the floor or ceiling. It has been demonstrated through previous research that the value of zero has noticeable effects when compared to other small, nonzero values (Zhang and Slovic, 2019), but it is unknown if the same effect, at the same rate, is observed with maximum values. To examine this, participants interacted with both an extreme- (gives three maximum or minimum offers) and a stable- (no maximum or minimum offers) behaving partner in a high offer (all offers above $5) or low offer (all offers below $5) condition. For both partners in the high and low conditions, the total mean amount of offers given was the same; the only difference was the range. Only extreme partners give the maximum or minimum; we are able to directly assess the impact of these offers when all other offers are similar in value. We then assessed their decision to reciprocate to each partner across the entire trust game and their subjective trust ratings of encountered partners.

The results indicated that extreme offers affect both reciprocity and trustworthiness, but more so when partners give low offers that are considered unfair. Although both partners in the high condition and low condition gave the same amount on average, the maximum of $10 and minimum of $0 in each condition appears to affect partner perception and reciprocations. This demonstrates that the actions in the trust game not only affected their behavior in the moment, but also left an impression on participants. Additionally, it is important to note that there was a significant interaction between high/low and extreme/stable conditions on trustworthiness ratings, but there was less evidence of this interaction during the task (i.e., in reciprocation behavior).

Specifically, there was a negligible difference between reciprocations and perceived trustworthiness in the high conditions, although the extreme-high partner was slightly more preferred. Therefore, although reciprocation did not vary much between partners in the high conditions, the maximum offers in the extreme-high condition slightly affected positive perception. Additionally, the extreme-low partner appeared to have a greater negative effect on both reciprocation and trustworthiness than both the extreme-high and stable-low partners. This suggests that extreme relative to stable offers may have less of an effect during real-time social interactions, but this effect may grow over time, skewing more negatively or positively when extreme actions are reflected on after the interaction. These results are both novel and important, giving much insight into how we behave in real-world social relationships in response to generosity and betrayal. Having collected data through Prolific, we were able to examine our research questions across the adult lifespan, and across diverse socioeconomic statuses and backgrounds. Therefore, we believe our nationally representative sample to yield validity of results.

### 4.1 Model 1 discussion: examining reciprocation across all trials and conditions

To evaluate reciprocation across the whole game, we conducted a repeated measures ANOVA to evaluate the rate of reciprocity in high and low and stable and extreme conditions, across six blocks of five trials. We hypothesized that reciprocation would be higher in the high condition compared to the low condition, and that extreme offers would have a larger impact on reciprocation rates in the low condition, leading to lower reciprocity in that condition. Through this analysis we found support for Hypothesis 1a, in that the between-subjects factor of high and low condition was a significant predictor of reciprocity. Our results also provided partial support for Hypothesis 1b. The within-subjects factor of extreme/stable partners was not a significant predictor of reciprocity. However, we observed significance in block, as well as the interaction between block and extreme and stable conditions, exhibiting changes in reciprocation as partner behavior is learned. Ultimately, there is not a significant difference in reciprocation behavior toward both partners in the high condition, while there is in the low condition. It is evident that participants are affected by extreme negative offers much more than extreme positive offers, modeled by reciprocations. This again supports our alignment with prospect theory in that gains do not substantially impact individuals as much as losses (Kahneman and Tversky, 1977). Additionally, the results support findings related to the zero effect, in that the value of zero is more substantial, even when compared to similar small, non-zero values (Zhang and Slovic, 2019).

### 4.2 Model 2 discussion: examining likelihood of reciprocation on last trial by condition

Model 2 examined participant behavior in the last trial of each block, and specifically their decision to reciprocate. This last choice does not reflect choices throughout the entire task but is a good indicator of learning as by this point, the participant has had 29 interactions with the partner. In theory, by the end of the game, participants should be fully conditioned to their partner's behavior and be making decisions in parallel to the degree of fairness they are receiving (Chang et al., 2010). Additionally, participants are expected to take the actual offer amount into account here, as the goal is to get the largest amount of money possible. We knew that, for obvious reasons, the participants in the high condition were going to be more likely to reciprocate than those in the low condition due to the simple fact of receiving all subjectively fair offers. We hypothesized that within-subjects, the consistently behaving partners would be more likely to receive a reciprocation in the last trial. Again, this hypothesis was formulated based on findings that humans tend to gravitate toward consistency in social relationships (Abbott et al., 1984). More specifically, offers appeared to be more consistently higher from the stable-high partner, as the $10 offers in the extreme-high condition brought relative offer amounts down to maintain the average. Additionally, we expected reciprocations to be more likely for the stable-low partner, since they appeared more trustworthy in that they had not given any $0 offers. The main hypothesis for Model 2 showed the model to be the best fit of the data, compared to the inverse and mixture models.

An interesting finding from Model 2 is that there was an almost negligible difference between reciprocation for both partners in the high condition (1.66%). This could be because all offers were above the median amount and could be considered favorable. This is consistent with the literature in that individuals tend to reciprocate simply because they are being reciprocated to in return (Chang et al., 2010). However, it is interesting that large offers of $10 found only in the extreme-high condition did not cause a substantial shift to trust behavior. Again, this could be due to the stable-high partner's offers appearing to be consistently higher due to the shift in average of the extreme-high offers caused by the $10. Previous studies have investigated the efficacy of promises and apologies to redeem broken trust in economic models and have found that they are relatively effective in the context of economic decision paradigms (Schniter et al., 2013). Although the current study did not include promises or apologies for unfair offers, a generous maximum offer is essentially doing the same thing: trying to make up for unfair behavior (less optimal offers, more frequently). However, again, although reciprocation was slightly higher, this did not have a significant effect on behavior. Overall, the results from Model 2 do support prospect theory (Kahneman and Tversky, 1977) in that the $0 (minimum) offers appeared to be more impactful than the $10 (maximum) offers, as shown by the differences in reciprocations between within-subjects conditions. These results are interesting because they exactly mirror those of Models 1 and 3, even though Model 2 only looked at behavior during the last choice.

### 4.3 Model 3 discussion: examining partner trustworthiness ratings across conditions

In Model 3, we examined trustworthiness ratings for each partner. We found support for Hypothesis 3a in that the significant main effect of high/low condition mirrored the results from Hypothesis 1a. Therefore, high conditions resulted in both higher trustworthiness ratings and more reciprocations. Again, we observed just a slightly higher average rating for extreme-high compared to stable-high partners, while the gap between ratings in the low conditions was greater. We also observed a significant interaction between high/low conditions and extreme/stable partners, both supporting Hypothesis 3b and mirroring results from Hypothesis 1b. Participants prefer the extreme-high partner slightly more, while there is a stronger preference toward the stable-low partner in the low condition. This indicates that the $0 offers in the extreme-low condition had a greater impact on trust than the $10 offers in the extreme-high condition. Even though trustworthiness ratings between both partners in the high condition were minuscule, participants preferred the extreme-high partner more, most likely due to the maximum $10 offers.

By the end of the task, participants had interacted with the partner in 30 trials and should have developed an opinion on the trustworthiness of that partner. This is observed to occur and appears to be in line with the recency effect (Rigdon et al., 2007). These results tell us much about how our actions and perceptions do align well, and extreme offers do tend to affect these domains. In both high (to a slight degree) and low domains, just a few actions can affect how we think about our social partners and act toward them. These results are also in line with prospect theory, in that the $0 offers were more negatively impactful than the $10 were positively impactful on trust (Kahneman and Tversky, 1977). These results further highlight the idea that zero has a notable effect, especially in comparison to other nonzero values (Palmeira, 2011).

### 4.4 Limitations

Although the current study yielded interesting results, we do have some limitations to note. First, we cannot determine how engaged participants were during completion of the study. This is always a risk with studies conducted in an online format, as there is no way to ensure that full attention is being exerted into the game play. Also, with Prolific as the platform of choice, participants were compensated to complete the study. The guaranteed payment may have influenced participants' motivation, either reducing strategic engagement due to lack of financial stakes or allowing them to experiment more freely without fear of loss. Participants were told that they would receive a bonus amount based on the outcome of a random trial, but it is not clear as to how much this actually affected their decisions during the game.

Another limitation is that our experiment did not explicitly inquire about the believability of the social cover story. Future research can address this limitation through funnel debriefing believability verification. As a compensatory design strength, however, our experiment involved real financial stakes. Participants were told that their decisions in the game would affect their real monetary bonus. Therefore, even if participants did not believe that they were interacting with another human being, they were explicitly told that they would receive a bonus based on their choices. Harrison and Ross (2017), leaders in the field of economic decision-making, strongly argue that experiments that include consequential decisions have a greater degree of validity than those with hypothetical decisions. Additionally, a review by Thielmann et al. (2021) concluded that economic games that include financial stakes are a more valid representation of choice behavior than those without. Therefore, even if participants did not believe that they were interacting with another human being, the study was designed to elicit incentive-compatible choices to allow for generalization to real-world behavior.

Despite any limitations, the significant patterns in our results indicate that these constraints did not undermine our key findings. For example, it could be suggested that our extreme conditions were not that extreme. It may be plausible to test the impact of true outlier amounts, making the maximum and minimum more extreme in future studies. The minimums and maximums in stable conditions were not far removed from $0 and $10. However, we argue that receiving $0 is much different from receiving even $1, as you are effectively getting *nothing*. Similarly, in the high condition, receiving the maximum possible offer has a greater impact than a dollar or so less. We also argue that incorporating more extreme outliers would potentially skew the results. We focused on creating an experimental design that was ecologically valid and translatable to the real world, where the effects of experimental conditions could be observed without ceiling or floor effects on reciprocity or perceived trustworthiness. In our social relationships, it is rare to experience truly extreme betrayal or generosity. In other words, we wanted to create a situation similar to what could occur in real life. When contemplating potential generous actions, for example, a friend would be more likely to buy your dinner than buy you a house. We feel that our study design accurately reflects behaviors in real-world situations, although it would be interesting to explore how greater extremities affect this behavior.

### 4.5 Conclusions

The current study's findings align with prospect theory, demonstrating that losses are given more weight than gains, and participants focus on loss aversion. This pattern is consistent across all models. Analyzing reciprocation across all trials and average trust ratings reveals the unique impact of extreme offers. Interestingly, although participants appeared to slightly prefer the extreme-high partner over the stable-high partner, the difference was marginal. While the maximum ($10) did have some effect, fair behavior was generally well received. We did, however, observe a greater disparity in the low condition. The extreme-low partners received fewer reciprocations and lower trust ratings compared to stable-low partners. Despite the scarcity of the $0 offers (occurring only three times out of thirty trials), they had a notable impact, consistent with previous findings related to the zero effect. These findings support the effect of zero beyond its function as a minimum value, indicating that other extreme-low values do not yield the same effects as zero. For example, the present study's findings add to recent work on the choices people make when faced with life-saving decisions (Zhang and Slovic, 2019). This prior study involved multiple conditions, each presenting a 50/50 probability that a variable number of individuals would either lose their lives or be saved. Across a variety of different contexts, participants made notably distinct decisions for scenarios involving lives lost vs. lives saved, and this domain effect was further qualified by a distinct effect observed for scenarios with exactly zero lives lost compared to other very small numbers of lives lost. Our study adds to this literature, demonstrating that trust-related decision-making is particularly affected by monetary offers of exactly zero from a social partner, compared to other small offers and extreme-high offers. In the context of economics, research on the uniqueness of zero has focused on consumers' choices in purchasing low-priced or free items (Shampanier et al., 2007; Gans, 2022). Aligned with our findings, an item priced at exactly zero dollars (i.e., free) compared to other low-priced items notably shifts behaviors (Shampanier et al., 2007).

While loss aversion may explain the observed asymmetry in response to extreme offers between high and low conditions, the relatively weak effect of extreme maximum offers, relative to minimum offers, is also consistent with research on the saturation of positive outcomes and the salience of generosity. The term saturation of positive outcomes is used to describe how incremental increases in already positive payoffs fail to meaningfully alter behavior (Mourali and Pons, 2009) because additional gains may carry diminished psychological weight when outcomes are already favorable. Furthermore, our finding that extreme vs. stable offers in the high conditions did not have distinctive effects on behavior or subjective trustworthiness may be due to salience of generosity, as all offers in the high condition were advantageous. Research on the salience of generosity (Brañas-Garza et al., 2017) suggests that our findings in the high conditions may be explained by participants failing to distinguish particularly generous behavior from generally fair behavior, since there is a normative expectation for such generosity.

Complimentary to theoretical implications, findings from the current study also have implications for real-world social interactions. The present research indicated that participants were less inclined to act fairly toward a partner who gave them a minimum ($0) offer. Although the differentiation between extreme and stable partners in the low condition was subtle, the effect is ultimately robust in terms of effects on trust perceptions. These findings highlight the lasting impact of extreme actions on trust, with extreme betrayal weighing more heavily than extreme generosity. This has relevance for relationships, workplace dynamics, and consumer protection programs. Specifically, our findings highlight how extreme actions of betrayal can have a lasting psychological impact, while extreme acts of generosity are not distinguished from consistent, lower magnitude acts of generosity.

## Data Availability

The raw data supporting the conclusions of this article will be made available by the authors, without undue reservation.
