# Supplementary material for: Effects of maximum and minimum offers on reciprocity and trust perceptions during economic decision-making
Source: Front Cognit. 2025 Nov 4;4:1576987. doi: 10.3389/fcogn.2025.1576987 (PMC13281041; doi:10.3389/fcogn.2025.1576987)
Supplement: Supplementary file 1 [file Table_1.docx]

Supplementary Material

# Supplementary Figures

**Supplementary Figure 1.** Non-social, neutrally-rated stimuli from the International Affective Picture System (IAPPS) used as partner avatars.


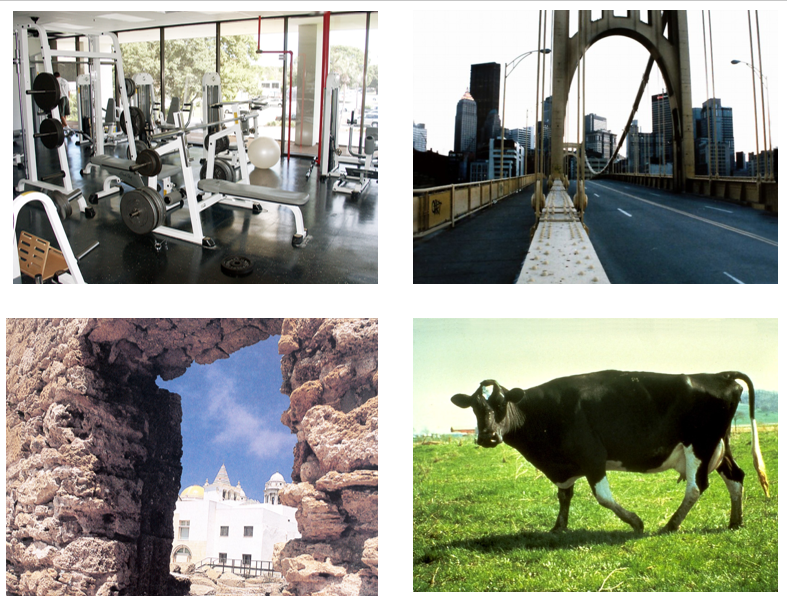


# Supplementary Tables

**Supplementary Table 1.** Output from Model 1; reciprocity means across blocks, stable/extreme and high/low conditions, with confidence intervals.

| **Variable** | ***M*** | ***SEM*** | ***95% CI, Lower*** | ***95% CI, Upper*** |
| --- | --- | --- | --- | --- |
| Low*Extreme*Block 1 | 2.85 | 0.20 | 2.45 | 3.25 |
| Low*Extreme*Block 2 | 2.80 | 0.19 | 2.43 | 3.18 |
| Low*Extreme*Block 3 | 2.95 | 0.20 | 2.57 | 3.34 |
| Low*Extreme*Block 4 | 2.94 | 0.19 | 2.57 | 3.31 |
| Low*Extreme*Block 5 | 2.68 | 0.20 | 2.29 | 3.07 |
| Low*Extreme*Block 6 | 2.54 | 0.20 | 2.15 | 2.93 |
| Low*Stable*Block 1 | 3.00 | 0.17 | 2.66 | 3.34 |
| Low*Stable*Block 2 | 2.94 | 0.20 | 2.53 | 3.34 |
| Low*Stable*Block 3 | 2.64 | 0.21 | 2.23 | 3.04 |
| Low*Stable*Block 4 | 2.56 | 0.20 | 2.16 | 2.96 |
| Low*Stable*Block 5 | 2.76 | 0.21 | 2.33 | 3.18 |
| Low*Stable*Block 6 | 2.64 | 0.20 | 2.24 | 3.04 |
| High*Extreme*Block 1 | 3.88 | 0.21 | 3.46 | 4.30 |
| High*Extreme*Block 2 | 4.12 | 0.20 | 3.72 | 4.51 |
| High*Extreme*Block 3 | 3.98 | 0.21 | 3.58 | 4.39 |
| High*Extreme*Block 4 | 3.93 | 0.20 | 3.54 | 4.32 |
| High*Extreme*Block 5 | 3.91 | 0.21 | 3.51 | 4.32 |
| High*Extreme*Block 6 | 3.97 | 0.21 | 3.56 | 4.38 |
| High*Stable*Block 1 | 4.28 | 0.18 | 3.92 | 4.64 |
| High*Stable*Block 2 | 4.07 | 0.22 | 3.64 | 4.49 |
| High*Stable*Block 3 | 3.95 | 0.22 | 3.52 | 4.38 |
| High*Stable*Block 4 | 3.93 | 0.21 | 3.51 | 4.36 |
| High*Stable*Block 5 | 3.95 | 0.22 | 3.50 | 4.39 |
| High*Stable*Block 6 | 3.88 | 0.21 | 3.46 | 4.30 |
